# ARPC1B Promotes Clear Cell Renal Cell Carcinoma Progression via the Wnt/β-Catenin Signaling Pathway

**DOI:** 10.32604/or.2025.067340

**Published:** 2025-09-26

**Authors:** Jiayin Peng, Yijun Xue, Zhiren Cai, Zhaoguan Li, Kangyan Han, Xiaoqi Lin, Yutong Li, Yumin Zhuo

**Affiliations:** 1 Department of Urology, The First Affiliated Hospital of Jinan University, Guangzhou, 510620, China; 2 Department of Urology, Central People’s Hospital of Zhanjiang, Guangdong Medical University, Zhanjiang, 524000, China

**Keywords:** Clear cell renal cell carcinoma (ccRCC), biomarker, epithelial-to-mesenchymal transition (EMT), Wnt/β-catenin signaling, actin-related protein 2/3 complex subunit 1B (ARPC1B)

## Abstract

**Background:**

Clear cell renal cell carcinoma (ccRCC) is an aggressive malignancy associated with limited treatment options and poor prognosis. Emerging studies suggest that the actin-regulating protein actin-related protein 2/3 complex subunit 1B (ARPC1B), a key regulatory protein within the actin cytoskeleton, could play a pivotal role in ccRCC progression. The current study aimed to uncover the biological functions of ARPC1B and the molecular mechanisms driving its effects in ccRCC.

**Methods:**

ARPC1B expression and prognostic implications were analyzed using data sourced from the Gene Expression Profiling Interactive Analysis (GEPIA) platform, immunohistochemical (IHC) staining on 150 tumor samples along with 30 corresponding normal tissues, and Western blotting (WB) analyses across multiple ccRCC-derived cell lines. Functional assays assessing cell proliferation, colony formation capability, migration, invasion, and *in vivo* tumorigenicity were conducted following either ARPC1B suppression or upregulation. Additionally, WB analysis was utilized to evaluate proteins linked to epithelial-to-mesenchymal transition (EMT) and the Wnt/β-catenin pathway.

**Results:**

The findings revealed a substantial elevation of ARPC1B in ccRCC tissues and cell lines, significantly associated with advanced TNM stages, higher Fuhrman grades, and reduced overall survival (OS) (*p* < 0.001). Multivariate statistical analysis identified ARPC1B as a standalone prognostic factor. Silencing ARPC1B notably impaired ccRCC cellular activities, and tumorigenesis in animal models, whereas augmented ARPC1B expression enhanced these malignant phenotypes. Mechanistically, downregulation of ARPC1B suppressed Wnt/β-catenin signaling and disrupted EMT, indicated by reduced β-catenin, c-Myc, cyclin D1, and ZEB-1 levels, and concurrently increased E-cadherin expression. Additionally, reactivation of the Wnt/β-catenin pathway partly reversed the inhibitory effects of ARPC1B depletion on tumor growth and invasiveness.

**Conclusions:**

ARPC1B emerges as an essential oncogenic factor in ccRCC by stimulating EMT and activating the Wnt/β-catenin pathway, ultimately enhancing tumor aggressiveness and metastatic potential. Thus, targeting ARPC1B represents a promising therapeutic strategy, warranting further exploration in ccRCC management.

## 1 Introduction

RCC is among the most prevalent and aggressive cancers involving the urinary system [1–3]. ccRCC constitutes about 80% of RCC diagnoses [4–7]. At initial diagnosis, nearly 30% of ccRCC patients already present with distant metastases [8,9], leading to a dismal 5-year survival rate of approximately 28% [10,11]. Surgical removal remains the most effective therapeutic approach for localized RCC, achieving survival outcomes as high as 97.4% for patients treated with partial nephrectomy at an early stage [12–14]. Thus, early diagnosis is essential for preventing metastasis and associated complications. However, ccRCC typically presents asymptomatically during early stages, and effective biomarkers for early detection remain unavailable. Although previous studies have identified various molecular markers, including DNA methylation [15–17], microRNAs [18–20], and long non-coding RNAs (lncRNAs) [21–23], their clinical application remains challenging due to the complexity of the molecular regulatory networks involved. Consequently, identifying reliable predictive biomarkers and clarifying their biological roles are critical steps toward early diagnosis and personalized treatment.

ARPC1B is an integral component of the ARP2/3 complex [24], characterized by structural similarities at its amino-terminal and carboxy-terminal ends [24]. ARPC1B significantly modulates actin cytoskeleton rearrangements and engages extensively with numerous proteins [25,26]. ARPC1B plays significant roles in various cellular processes, particularly in regulating cytoskeletal remodeling, cell motility, and invasion [27]. Additionally, ARPC1B participates in cell division processes and modulates multiple signaling pathways [26,28]. Elevated ARPC1B expression has been reported in various cancers, correlating with increased malignancy, invasiveness, and poorer clinical outcomes in prostate cancer [29], ovarian cancer [30,31], and glioblastoma [32,33]. However, its specific biological function and mechanism in ccRCC have not been thoroughly explored.

This study aims to investigate the clinical significance of ARPC1B expression in ccRCC and elucidate its functional contributions to tumor progression. We hypothesize that ARPC1B promotes ccRCC aggressiveness through activation of the Wnt/β-catenin signaling pathway and induction of epithelial-mesenchymal transition. Utilizing comprehensive *in vitro* and *in vivo* approaches, this research seeks to establish ARPC1B as both a prognostic biomarker and a potential therapeutic target for ccRCC management.

## 2 Materials and Methods

### 2.1 Database and Data Analysis

Gene Expression Omnibus (GEO; https://www.ncbi.nlm.nih.gov/geo/) (accessed on 14 August 2025) provided the transcriptome datasets GSE53757 and GSE68418 that were utilized in this study. This data was preprocessed in R/Bioconductor with the limma package. Precision weighting was accomplished using the voom method, normalization was based on quantiles, and background correction was accomplished using the Robust Multiarray Average (RMA) method. To find differentially expressed genes (DEGs), an empirical Bayes method was employed, using stringent criteria like a |log_2_ fold-change (log_2_ FC)| > 1 and an adjusted *p*-value False Discovery Rate (FDR) < 0.05. We accomplished functional enrichment for Gene Ontology (GO) keywords and Kyoto Encyclopedia of Genes and Genomes (KEGG) pathways using the package clusterProfiler (v4.0.5; Guangchuang Yu Lab, Guangzhou, China). The clinical validation of ARPC1B expression was investigated using GEPIA2 (http://gepia2.cancer-pku.cn/) (accessed on 14 August 2025).

### 2.2 Tissue Specimens and IHC Analysis

The American Joint Committee on Cancer (AJCC) 8th edition TNM staging system was employed for all cases. This tissue microarray (TMA; XT15-050) includes 150 ccRCC tissues from patients who underwent radical nephrectomy with regional lymph node dissection and 30 matched neighboring non-tumor controls; it was acquired from Shanghai Outdo Biotech Co., Ltd. (Shanghai, China) (SOBC). Critically, all surgical specimens had confirmed pathological nodal status (pN0/pN1) with no pNX cases, enabling precise pathological TNM staging (pT/pN). In the years 2008–2010, the pathological validity of surgical specimens was determined. Approval No. SHYJS-C1510001 from the SOBC Ethics Committee meant that the research could go forward in compliance with the 1975, revised 2000 Helsinki Declaration. Informed consent was obtained from all participants. For in-house histological detection of ARPC1B, Proteintech of Wuhan, China, utilized rabbit polyclonal antibodies (Cat# 13835-1-AP; dilution 1:2000). Sections were preheated at 60°C for 1 h before being rehydrated with graded ethanol (100%, 90%, and 70%, 5 min each concentration). The next step was deparaffinization twice in pure xylene, with a 15-min interval between each cycle. The sections were subjected to 3% hydrogen peroxide for 5 min to eliminate any intrinsic peroxidase activity. Once that was done, they were rinsed with phosphate-buffered saline (PBS; 0.01 M, pH 7.4; Sigma, St. Louis, MO, USA, Cat# P3813) that included 0.5% Tween 20. For 5 min at room temperature, DAKO/Agilent’s (Glostrup, Denmark) Cat# S2023, which is for protein blocking without serum, was used. To observe the results of the immunoreactivity test, antibodies were diluted with DAKO antibody diluent and then placed into the EnVision Plus Anti-Rabbit Labeled Polymer Kit (DAKO/Agilent, Cat# S0809). To ascertain the outcomes of the immunostaining procedure, the staining intensity and percentage of cells that tested positive were quantified. For no staining at all, a score of 0, moderate staining at 2, strong staining at 3, and a value of 4, indicating that more than 25% of cells stained positively, were all possible. To get the overall IHC score (ranging from 0 to 12), we multiplied the staining intensity by the percentage. With a score of 6 or more, ARPC1B expression was considered high, while with a score of 6 or lower, it was considered low. Three highly experienced uropathologists evaluated the materials separately, without any background in clinical practice.

### 2.3 Plasmids and Short Hairpin RNA (shRNA)

Lentiviral constructs for ARPC1B overexpression and gene silencing were obtained commercially (GeneChem Co., Ltd., Shanghai, China). To achieve overexpression (OE-ARPC1B), the complete human ARPC1B open reading frame (ORF, NM_005720.4) was cloned into a GV492 lentiviral vector (GeneChem, Cat# GV492). For ARPC1B silencing, three different shRNA sequences targeting ARPC1B were inserted into GV493 lentiviral vectors (GeneChem, Cat# GV493). These constructs included shRNA1 (#118761: 5^′^-GCTGACCTTCATCACAGACAA-3^′^), shRNA2 (#118762: 5^′^-GCTGGGTACATGGCGTCTGTT-3^′^), and shRNA3 (#118763: 5^′^-CCCAACGAGAACAAGTTTGCT-3^′^). A scrambled control shRNA (con#1: 5^′^-TTCTCCGAACGTGTCACGT-3^′^) was also synthesized. For knockdown experiments, GV493 vectors expressing scrambled shRNA sequences were transduced as negative controls (-NC), while empty GV492 vectors lacking ARPC1B cDNA sequences served as control vectors (-Vec) in overexpression experiments.

### 2.4 Cell Culture

The human ccRCC cell lines 786-O (ATCC CRL-1932; short tandem repeat (STR)-authenticated Dec/2022; Mycoplasma-free) and Caki-1 (ATCC HTB-46; STR-authenticated Nov/2022; Mycoplasma-free) were procured from Pricella Biotechnology Co., Ltd. (Wuhan, China). Additionally, ccRCC cell line A-498 (ATCC HTB-44; STR-authenticated Jan/2023; Mycoplasma-free), papillary renal carcinoma cell line Caki-2 (ATCC HTB-47; STR-authenticated Jan/2023; Mycoplasma-free), and normal renal tubular epithelial cell line HK-2 (ATCC CRL-2190; STR-authenticated Aug/2022; Mycoplasma-free) were generously provided by Professor Jian Wang (Guangdong Medical University, Zhanjiang, China). Standard conditions at 37°C and 5% CO_2_ were used for cell culture in RPMI-1640 medium (Gibco, Waltham, MA, USA, C11875500BT) with 10% fetal bovine serum (FBS) and penicillin-streptomycin solution (PS; Solarbio Technology, Beijing, China, P1400). Utilizing lentiviral-mediated gene delivery, stable ARPC1B overexpression and knockdown cell lines were established. These cell lines were engineered utilizing GV492 vectors encoding ARPC1B or GV493 vectors expressing particular shRNAs. The cells were treated with polybrene (8 μg/mL; Sigma, MO, USA, Cat# TR-1003-G) throughout the lentiviral infection. After that, they were cultured in RPMI-1640 media with 2 μg/mL puromycin (Thermo Fisher, MA, USA, Cat# A1113803) for 72 h.

### 2.5 Quantitative Reverse Transcription Polymerase Chain Reaction (RT-qPCR)

Isolating total RNA from different isogenic cell groups (786-O and Caki-1 parental, ARPC1B-overexpressing, ARPC1B-knockdown, and corresponding vector/scrambled control cells) was done using the TRIzol reagent (Thermo Fisher Scientific, Cat# 15596026). The ToloScript All-in-one RT kit (Tolobio, Shanghai, China, Cat# 22107)’s instructions were followed for the synthesis of complementary DNA (cDNA). The 2× SYBR Green Master Mix (Applied Biosystems, Thermo Fisher Scientific, Carlsbad, CA, USA, Cat# A46109) was used to conduct the real-time PCR. The ARPC1B amplification primers were 5^′^-CAAGGACCGCACCCAGATT-3^′^ and 5^′^-TGCCGCAGGTCACAATACG-3^′^. The internal control β-actin primers were 5^′^-TGGAACGGTGAAGGTGACAG-3^′^ and 5^′^-TTAGAGAGAAGTGGGGTGGC-3^′^. Using threshold cycle (Ct) values, the relative quantification of ARPC1B mRNA expression was computed using the 2^−ΔΔCt^ method.

### 2.6 Cell Proliferation Assay

Cell Counting Kit-8 (CCK-8; Dojindo, Tokyo, Japan, Cat# CK04) was used to evaluate the cells’ performance. So that rescue studies could be carried out, cells that did not have ARPC1B were treated for 24 h before plating with 30 μM of Wnt/β-catenin agonist 3 (Proteintech, Wuhan, China, Cat# CM20349). Proliferation was then assessed at 0, 24, 48, 72, and 96 h after cell seeding at 5,000 cells/well in 96-well plates using 786-O and Caki-1 isogenic panels. Each well was incubated for an additional two hours at 37°C with 5% CO_2_ after ten microliters of CCK-8 solution were added at each time interval. The data was acquired from an optical density (OD) of 450 nm using a Synergy H1 microplate reader (BioTek, Winooski, VT, USA).

### 2.7 Colony Formation Assay

After being seeded onto 6-well plates from the isogenic 786-O and Caki-1 panels in triplicate at around 500 cells per well, cells were cultured continuously for 12 days. The colonies were successfully stained with a 0.5% crystal violet solution and then fixed in 100% methanol. If a colony lacked at least 50 cells, it was not counted using the ImageJ software (v1.53; NIH, Bethesda, MD, USA) program. The colony formation rate was calculated using the formula: (Number of colonies/500 seeded cells) × 100%.

### 2.8 Wound Healing Assay (WHA)

During the whole experiment period, the ARPC1B-knockdown cells were consistently treated with 30 μM of Wnt/β-catenin agonist 3 to create relief circumstances. In 6-well plates, cells from the 786-O and Caki-1 groups were grown for 24 h after being seeded at 1 × 10^5^ cells/well in order to assess migratory capacity. We then used a sterile 1 mL pipette tip to make a linear scratch in the cell monolayer. After that, we gently washed the cells to remove any detached cells. A Nikon Eclipse Ts2 inverted fluorescent microscope (Nikon Instruments Inc., Minato-ku, Tokyo, Japan) was used to take photographs of the wound closure at 0, 24, 48, and 72 h. At each specified time point, the migrated area was quantified using the ImageJ program.

### 2.9 Transwell Assays

The rescue tests involved supplementing both chambers of the transwell device with 30 μM of Wnt/β-catenin agonist 3. The migration assays were conducted by adding 5 × 10^4^ cells from the 786-O and Caki-1 isogenic groups to the upper chamber of a 24-well transwell insert (manufactured by Jet Biotherapy, Guangzhou, China) with an 8.0 μm pore. The cells were then suspended in 200 μL of serum-free RPMI-1640 culture. The 500 μL lower chambers were supplemented with 10% FBS. In order to investigate invasion, matrix gel (MCE, Monmouth Junction, NJ, USA, Cat# HY-K0301) was utilized before 5 × 10^4^ cells were seeded into the upper chambers. The cells that managed to pass the membrane throughout the 16-h incubation period were carefully preserved in 100% methanol and stained with 0.5 percent crystal violet. Five randomly chosen microscopic fields per chamber were captured at a 200× magnification, and then the number of cells that moved or invaded was automatically tallied using ImageJ software.

### 2.10 WB Assay

The proteins from ccRCC tissues or cells from the isogenic 786-O and Caki-1 panels were extracted using radioimmunoprecipitation assay (RIPA) buffer (Solarbio, Beijing, China, Cat# R0020) that contained protease and phosphatase inhibitors (Roche, Basel, Switzerland, Cat# 04693159001). Protein levels were quantified using the BCA assay (Cat# 23225; Pierce, Waltham, MA, USA). Electrophoretic conditions of 80 V for 30 min and 120 V for 45 min were used to separate samples with 30 μg of protein per lane. The samples were denatured in 4× Laemmli loading buffer (Bio-Rad, Hercules, CA, USA, Cat# 1610747). For complete protein transfer, intact gels containing pre-stained molecular weight markers (10–250 kDa) were electrophoretically transferred to PVDF membranes (0.45 μm; Millipore, Burlington, MA, USA, Cat# IPVH00010) using a wet transfer system at constant 200 mA current. Transfer durations were optimized according to protein molecular weight: high-molecular-weight proteins (e.g., ZEB-1 at 210 kDa) required extended transfer times of 120 min to ensure efficient membrane immobilization, while standard-duration transfers of 45 min were employed for mid-range molecular weights, including β-actin (42 kDa) and ARPC1B (41 kDa). The blocking process was carried out for 1 h using 5% skim milk in Tris-buffered saline with Tween-20 (TBST, pH 7.6). Following an overnight primary antibody incubation period at 4°C, the membranes were subjected to three five-minute washes with TBST. After that, primary antibodies (1:10,000; goat anti-rabbit/mouse; Abcam, Cambridge, UK, Cat# ab6721/ab6789) that were conjugated with HRP were left to incubate at 4°C for 1.5 h. In order to identify protein signals, researchers utilized enhanced chemiluminescence (ECL; Bio-Rad, Hercules, CA, USA, Cat# 1705060). Each experiment was repeated three times independently.

### 2.11 Antibodies and Reagents

Antibodies utilized in this research included: β-actin (Cat# 66009-1-Ig; WB 1:10,000), ARPC1B (Cat# 13835-1-AP; WB 1:5,000, IHC 1:1000), c-Myc (Cat# 10828-1-AP; WB 1:5,000, IHC 1:2000), Cyclin D1 (Cat# 60186-1-Ig; WB 1:10,000, IHC 1:3000), GSK3β (Cat# 22104-1-AP; WB 1:5,000, IHC 1:400), Phospho-GSK3β (Ser9) (Cat# 14850-1-AP; WB 1:5,000, IHC 1:600), β-catenin (Cat# 51067-2-AP; WB 1:10,000, IHC 1:200), Vimentin (Cat# 60330-1-Ig; WB 1:10,000, IHC 1:3000), N-cadherin (Cat# 22018-1-AP; WB 1:6000, IHC 1:1000), E-cadherin (Cat# 20874-1-AP; WB 1:6000, IHC 1:100), ZEB1 (Cat# 21544-1-AP; WB 1:2000, IHC 1:100), and Ki-67 (Cat# 27309-1-AP; IHC 1:2000) can from Proteintech. The Wnt/β-catenin agonist 3 (Cat# CM20349, 30 µmol/mL) was obtained from Proteintech (Wuhan, China, Cat# CM20349).

### 2.12 Tumor Xenograft Model

Hunan Slaike Jingda Experimental Animal Co., Ltd. of Changsha, China, was the source of the 24 male BALB/c-nu mice. License No. SCXK 2021-0002 certifies that these 4-week-old, 18–22 g specimens are of SPF grade. The mice were acclimated to their new environment for one week before being employed in the research. No infections were present in this environment. Stratified randomization was used to divide the mice into four groups, with six mice in each, after they had acclimated. To inject subcutaneously into the axillary region, cells (5 × 10^6^ cells per animal), particularly 786-O/shARPC1B#1 or Caki-1/shARPC1B#1, were mixed with 100 μL of PBS and Matrigel (Corning, Bedford, MA, USA, Cat# 356234; 1:1 ratio v/v). Tumor dimensions were measured every 48 h by two separate investigators who were blinded to the therapy groups, starting seven days after injection. (L × W^2^)/2 was used to determine the tumor volume. Ethical endpoints, such as tumor size > 1500 mm^3^ or body weight loss > 20%, or day 14 post-inoculation, were used to euthanize the animals. Isoflurane 5% (RWD Life Science, Shenzhen, China), administered in 100% oxygen and maintained at a dosage of 2.5% was used to induce anesthesia during the cervical dislocation procedure. Upon tumor collection, they were assessed for size, mass, and fixation in 4% neutral buffered formalin (Cat# HT50128) from Sigma-Aldrich in St. Louis, MO, USA for 24 h. After that, paraffin was used to embed them. Deparaffinization was performed on the 4 µm thick sections using xylene and graded ethanol. Prior to conducting the subsequent immunohistochemistry (IHC) studies, the samples were stained with Mayer’s hematoxylin (Sigma-Aldrich, St. Louis, MO, USA, Cat# MHS16; 0.1% w/v) and eosin (Sigma-Aldrich, St. Louis, MO, USA, Cat# HT110116; 1% w/v) according to standard procedures [34]. Zhanjiang Central People’s Hospital’s Animal Research Committee gave their stamp of approval to all animal procedures (Approval No. DW-2024016-01, Zhanjiang, China) and ensured that they adhered completely to the 3R principles: replacement, reduction, and refinement.

### 2.13 Statistical Analysis

Statistical analyses were performed using SPSS software (IBM, Armonk, NY, USA, version 27.0). Comparisons of categorical variables were conducted utilizing Fisher’s exact tests or chi-square tests, especially in instances where the expected frequency in any cell fell below five. Based on the distribution characteristics of continuous variables, we employed either Mann–Whitney U tests, Student’s *t*-tests, or Kruskal–Wallis tests. The analysis of cell proliferation under different conditions was conducted using two-way repeated-measures ANOVA. Survival outcomes were assessed through the creation of Kaplan–Meier survival tests. The application of Cox proportional hazards regression was utilized for both univariate and multivariate evaluations. The threshold for statistical significance was established at *p* < 0.05.

## 3 Results

### 3.1 ARPC1B Overexpression in Tumor Tissues Correlates with Poor Prognosis of ccRCC

**Figure 1:**
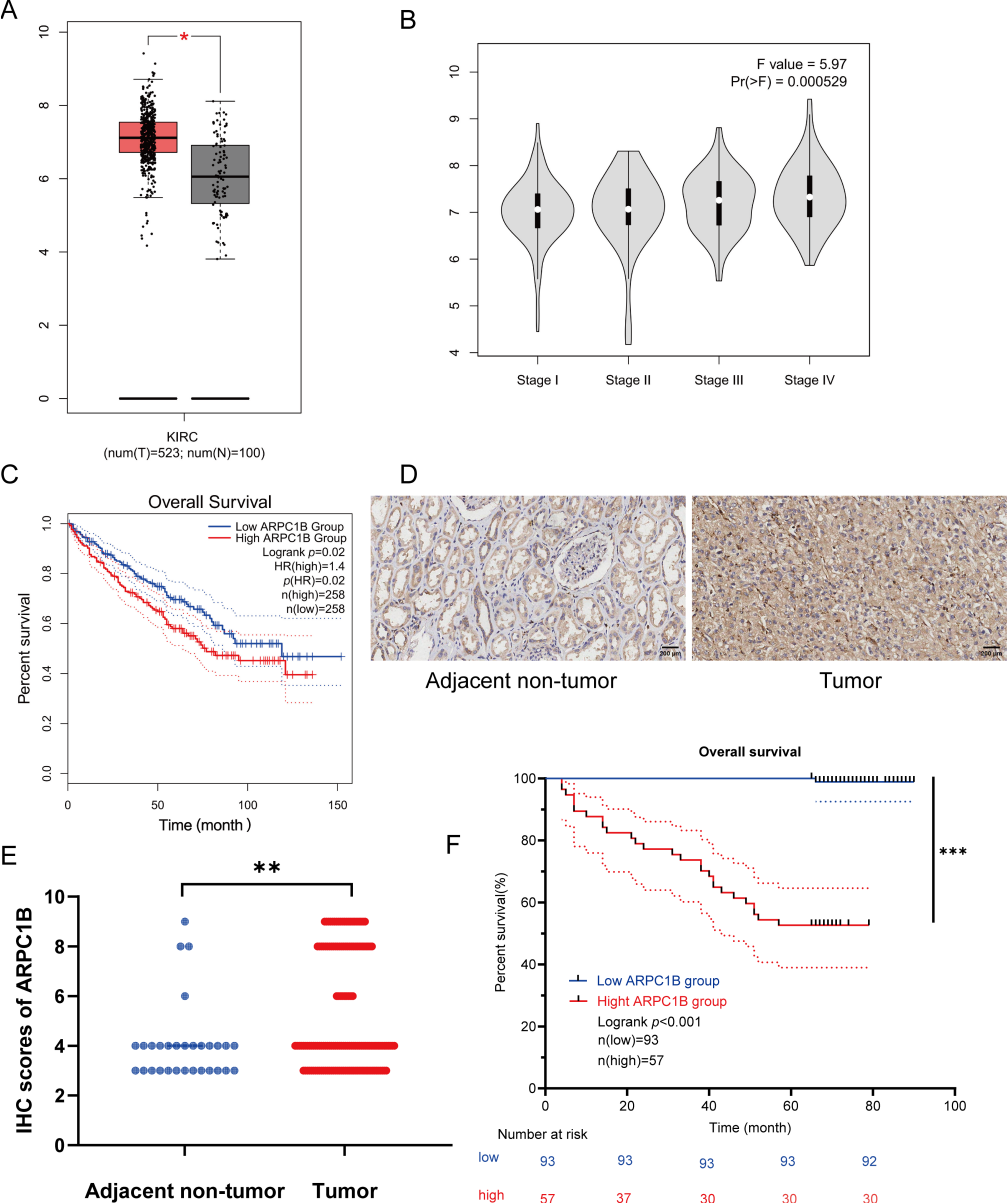
Enhanced ARPC1B expression in ccRCC tissues predicts poor patient prognosis. (**A**) ARPC1B expression across ccRCC samples from GEPIA (*Y*-axis: log_2_ [TPM + 1], TPM: transcripts per million). (**B**) ARPC1B expression stratified by tumor stage from GEPIA (*Y*-axis: log_2_ [TPM + 1]). (**C**) OS analysis based on median ARPC1B expression level; HR: hazard ratio. (**D**) Representative IHC images showing ARPC1B expression in ccRCC vs. normal adjacent tissues. (**E**) Quantitative comparison of IHC scores between ccRCC and non-tumor tissues. (**F**) Kaplan–Meier curves stratified by ARPC1B expression. *p < 0.05, **p < 0.01, ***p < 0.001

**Figure A1:**
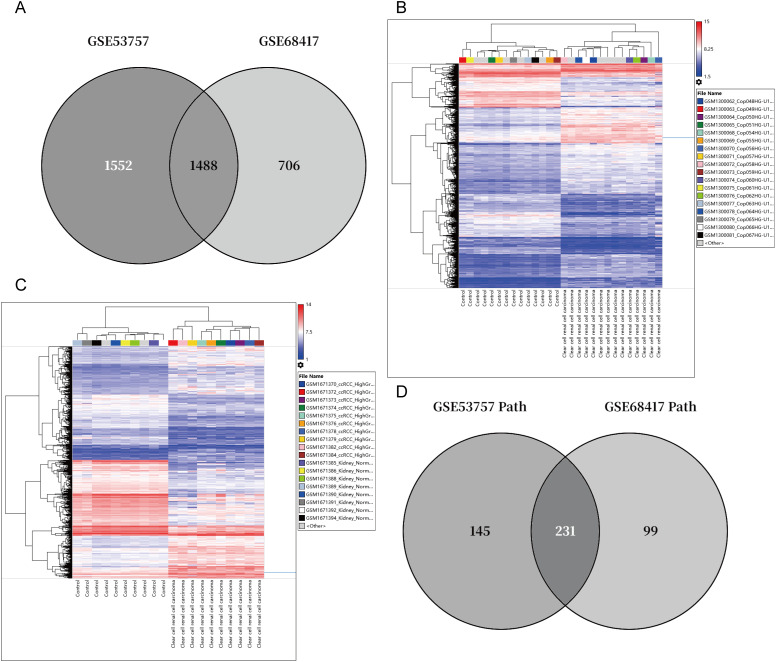

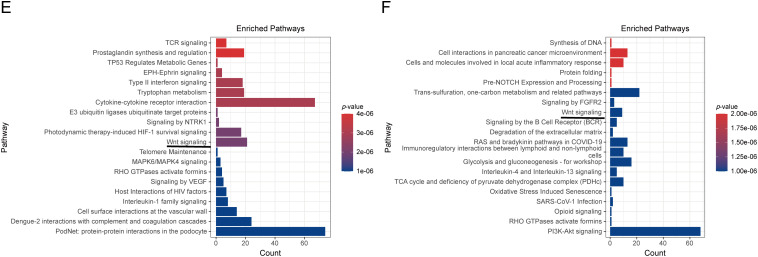
Cross-Cohort Transcriptomic Analysis of ccRCC. (**A**) Venn diagram showing 1488 overlapping differentially expressed genes (DEGs, |log_2_FC| > 1, FDR < 0.05) between GSE53757 (n = 3040 DEGs) and GSE68417 (n = 2194 DEGs). (**B,C**) Heatmap of GSE53757, GSE68417. The blue line indicates ARPC1B. (**D**) Pathway overlap analysis identifies 231 shared enriched pathways (FDR < 0.05) from 376 (GSE53757) and 330 (GSE68417) total pathways. (**E,F**) Top 10 enriched pathways in GSE53757 and GSE68417, including Wnt/β-catenin

**Figure A2:**
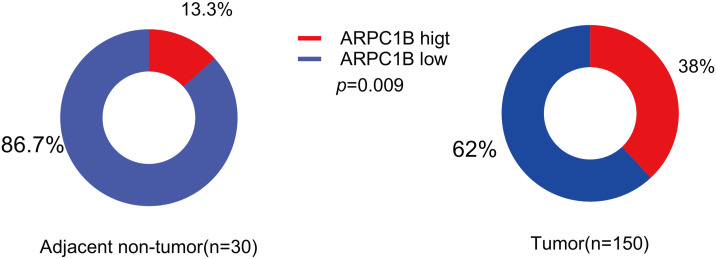
Levels of ARPC1B-positive (IHC score ≥ 6) in ccRCC tissues (n = 150) were much greater than in adjacent non-tumor tissues (n = 30) (38% vs. 13%, *p* = 0.009)

Transcriptomic analysis identified 1,488 differentially expressed genes (DEGs) common to both GEO datasets GSE53757 and GSE68417 (|log_2_FC| > 1, FDR < 0.05), including *ARPC1B* (Fig. A1A–C). Pathway enrichment via clusterProfiler revealed 231 conserved pathways (FDR < 0.05), with Wnt/*β*-catenin signaling demonstrating the most significant dysregulation (Fig. A1D–F). Complementing these findings, GEPIA analysis demonstrated significant overexpression of ARPC1B in ccRCC vs. normal kidney tissues (Fig. 1A). In addition, ARPC1B expression significantly increased with advancing clinical disease stage (*p* = 0.000529, Fig. 1B). Higher levels were related to worse OS (Fig. 1C). Expression of ARPC1B protein in 150 ccRCC specimens, including 30 matched non-cancerous samples, was analyzed by IHC staining. ARPC1B is predominantly localized to the cytoplasmic regions (Fig. 1D). Elevated ARPC1B was identified in 57 (38%) of the tumor samples, significantly higher compared to only 4 cases (13%) in matched non-tumorous tissues (OR = 0.2510; 95% CI: 0.09093–0.7292; *p* = 0.009; Fig. A2). Furthermore, quantitative scoring revealed notably increased ARPC1B protein levels within tumor tissues (mean ± SD: 5.3 ± 2.3) relative to the paired adjacent normal samples (4.1 ± 1.6; 95% CI: 0.3442–2.096; *p* < 0.01; Fig. 1E). ARPC1B expression exhibited significant relationships with clinical parameters, particularly tumor stage (T stage; *p* = 0.003) and Fuhrman grading (*p* < 0.001). However, no clear relationship was found between ARPC1B levels and patient demographics such as age, sex, or lymphatic metastasis (all *p* < 0.05), as illustrated in Table 1. Survival curves generated by Kaplan–Meier analysis indicated that patients expressing higher ARPC1B had notably worse OS (*p* < 0.001; Fig. 1F). Similarly, univariate Cox regression confirmed high ARPC1B as predictive of diminished survival (HR = 59.6; 95% CI: 8.1–439.1; *p* < 0.001; Table 2). Multivariate Cox regression further reinforced the independent prognostic significance of elevated ARPC1B (HR = 44.0; 95% CI: 5.8–334.5; *p* < 0.001; Table 2). Taken together, these observations robustly support the notion that ARPC1B elevation characterizes ccRCC and is associated with unfavorable clinical prognosis.

**Table 1:** Association between ARPC1B protein expression and clinicopathological characteristics in 150 ccRCC patients

Variable	Patients	No. of patients (%)		OR	95% CI	*p*
		ARPC1B Low (93)	ARPC1B High (57)			
Age, years (median 57)				1.488	0.767–2.889	0.239
<57	75	50	25			
≥57	75	43	32			
Sex				0.620	0.291–1.322	0.214
Male	107	63	44			
Female	43	30	13			
pTNM Stage				8.531	1.772–41.071	0.003^#**^
T1–T2	139	91	48			
T3–T4	11	2	9			
Lymph-node status						0.053^#^
N0	147	93	54			
N1	3	0	3			
Fuhrman Grade				6.656	3.110–14.246	0.001***
G1–G2	103	78	25			
G3–G4	47	15	32			

Note: # Using Fisher’s exact test; **p < 0.01; ***p < 0.001; ARPC1B, actin-related protein 2/3 complex subunit 1B; RCC, renal cell carcinoma.

**Table 2:** Cox regression analysis for OS

Variable	Univariate analysis			Multivariate analysis		
	HR	95% CI	*p*	HR	95% CI	*p*
pTNM stage						
T3–4 vs. T1–2	6.644	2.813–15.695	<0.001***	2.042	0.774–5.388	0.149
Lymph-node status						
N1 vs. N0	23.486	6.286–87.755	<0.001***	4.900	1.181–20.324	0.029*
Fuhrman grade						
G3–4 vs. G1–2	4.911	2.264–10.650	<0.001***	1.486	0.643–3.434	0.354
ARPC1B expression						
High vs. low	59.588	8.086–439.121	<0.001***	43.992	5.785–334.511	<0.001***

Note. *p < 0.05; ***p < 0.001; ARPC1B, actin-related protein 2/3 complex subunit 1B; HR, hazard ratio; CI, confidence interval.

### 3.2 Establishment of Stable Cell Lines and RT-qPCR Validation

**Figure 2:**
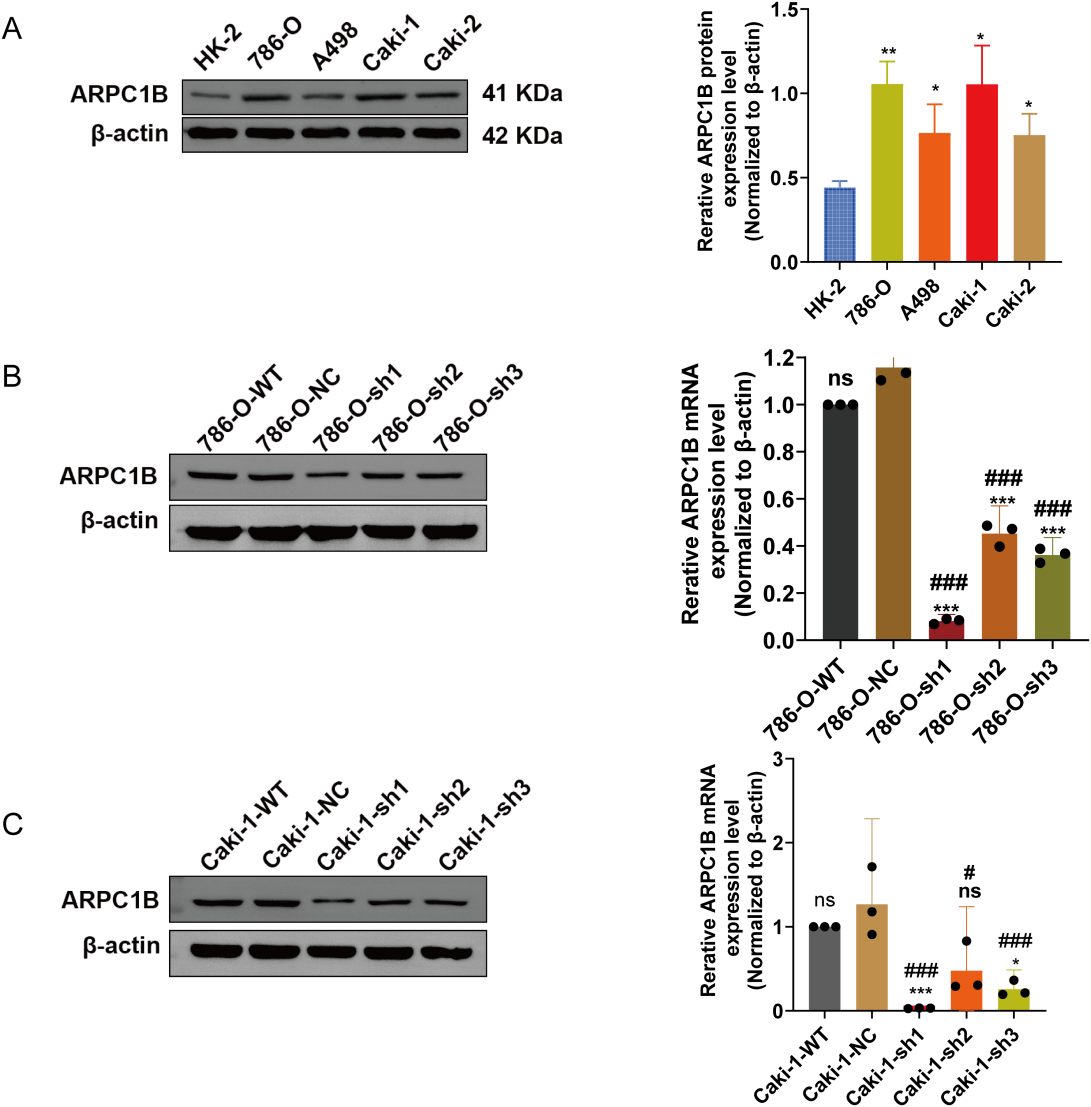
Validation of ARPC1B protein and mRNA levels in RCC cell lines. (**A**) WB analysis of ARPC1B expression in normal renal tubular epithelial HK-2 cells vs. RCC-derived cells. All samples were analyzed simultaneously under consistent conditions, and were normalized to β-actin. (**B,C**) Quantification of ARPC1B protein and mRNA in ARPC1B-knockdown cells (786-O and Caki-1) compared with WT and negative controls (-NC; GV493-scrambled). Compared to the sh-NC group; *p < 0.05, **p < 0.01, ***p < 0.001, ns, *p* **> **0.05; Compared to the WT group; ^#^*p* < 0.05, ^###^*p* < 0.001

**Figure A3:**
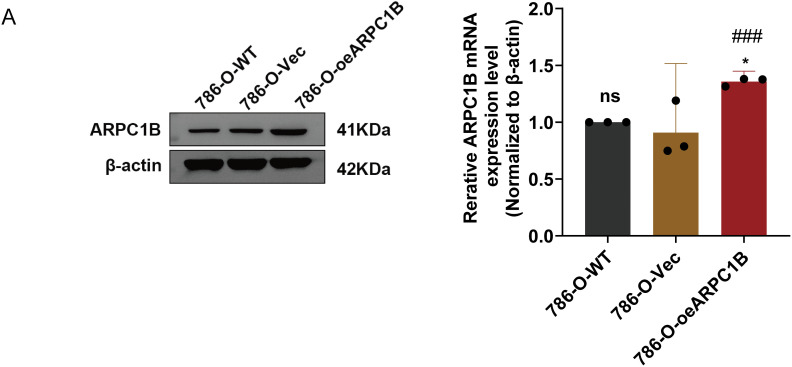

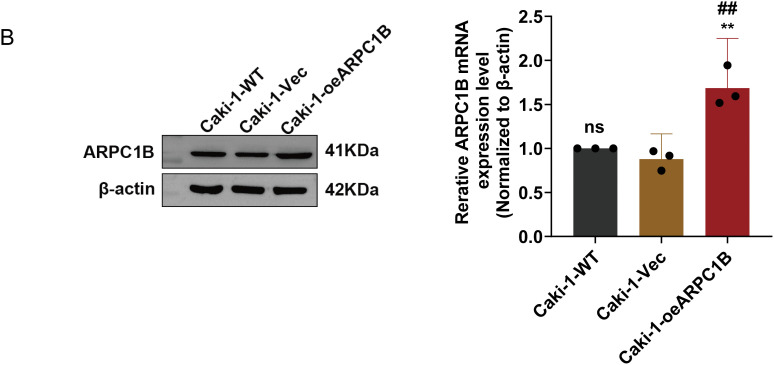
Validation of ARPC1B protein and mRNA levels in ccRCC cell lines. (**A,B**) Quantification of ARPC1B protein and mRNA in ARPC1B-overexpressing cells (786-O and Caki-1) compared with WT and negative controls (-Vec; GV492-empty). All samples were analyzed simultaneously under consistent conditions, and were normalized to β-actin. Compared to the Vec group, *p < 0.05, **p < 0.01, ns, *p >* 0.05. Compared to the WT group, ^##^*p* < 0.01, ^###^*p* < 0.001

There was a notable increase in the expression of the ARPC1B protein in all ccRCC-derived cell lines (786-O, A-498, and Caki-1) and papillary renal carcinoma cell line Caki-2 (*p* < 0.05; Fig. 2A). Among these, the cell lines 786-O and Caki-1 exhibited the most elevated levels of ARPC1B, leading to their selection for subsequent functional studies. Subsequently, stable cell lines with ARPC1B overexpression and knockdown were created from these ccRCC cell models. Successful ARPC1B knockdown was confirmed by RT-qPCR and WB, showing significant reduction of ARPC1B protein in knockdown groups compared with both negative control (NC) and wild-type (WT) cells (*p* < 0.001; Fig. 2B for 786-O; Fig. 2C for Caki-1). Conversely, ARPC1B overexpression was validated by increased ARPC1B levels relative to the corresponding WT and empty vector (Vec) controls (*p* < 0.05 for both cell lines; Fig. A3A,B). Quantitative analysis showed that ARPC1B protein abundance in overexpressed cells was approximately twofold greater than endogenous levels. Collectively, these results confirm the generation of robust ARPC1B-knockdown and -overexpression cell models, reinforcing the elevated expression of ARPC1B in ccRCC and its potential oncogenic role.

### 3.3 ARPC1B Knockdown Inhibits ccRCC Cellular Functions In Vitro

**Figure 3:**
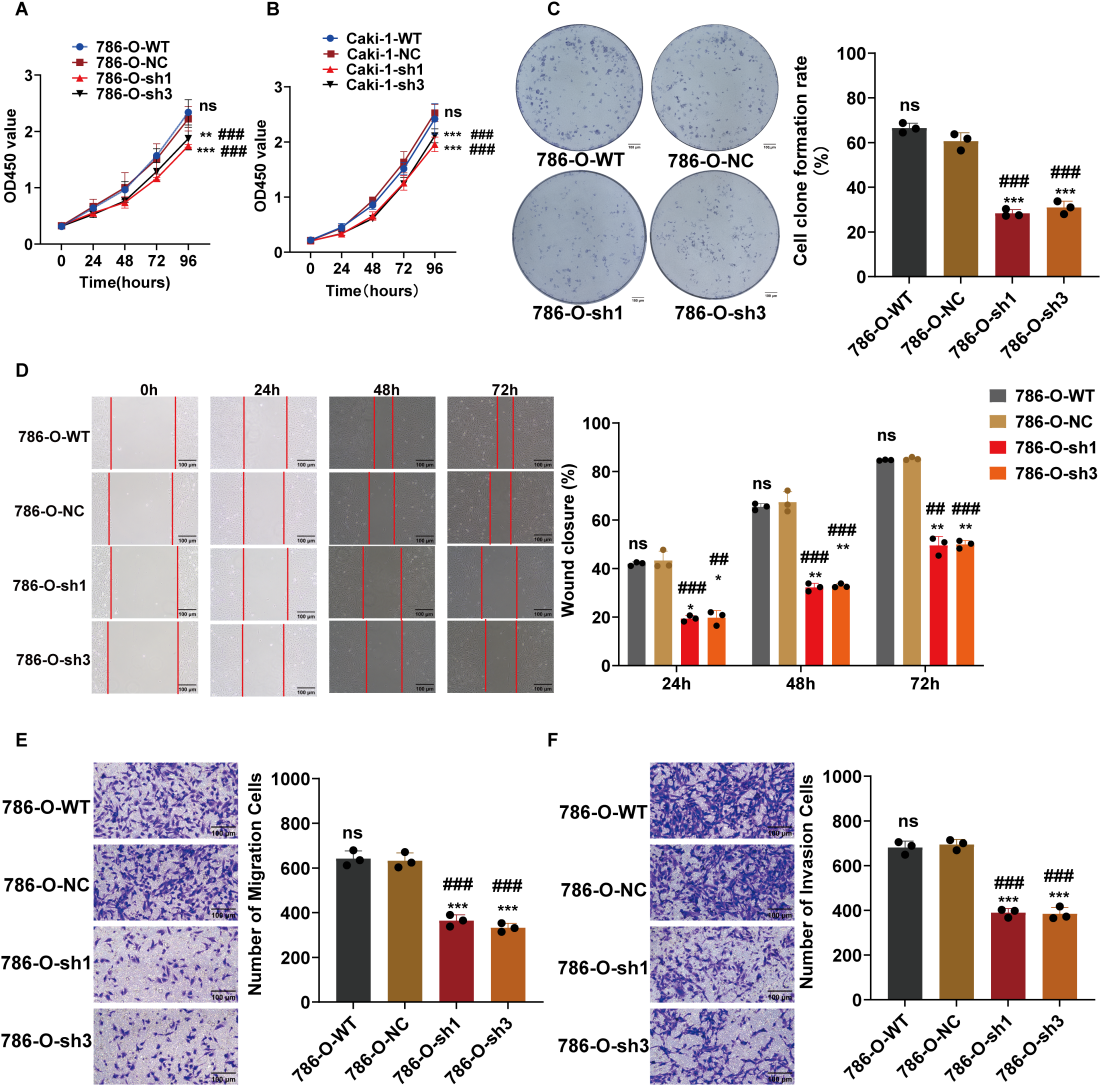
Functional effects of ARPC1B silencing on ccRCC cells. (**A,B**) CCK-8 showing decreased growth rates in ARPC1B-knockdown 786-O and Caki-1 cells. (**C**) Reduced clonogenic potential of 786-O cells after ARPC1B knockdown. (**D**) Inhibition of migration observed in 786-O cells upon ARPC1B silencing (WHA). (**E,F**) Reduced migration and invasion capabilities demonstrated by Transwell assays following ARPC1B knockdown. Compared to the sh-NC group; *p < 0.05, **p < 0.01, ***p < 0.001, ns, *p* **> **0.05. Compared to the WT group; ^##^*p* < 0.01, ^###^*p* < 0.001

**Figure A4:**
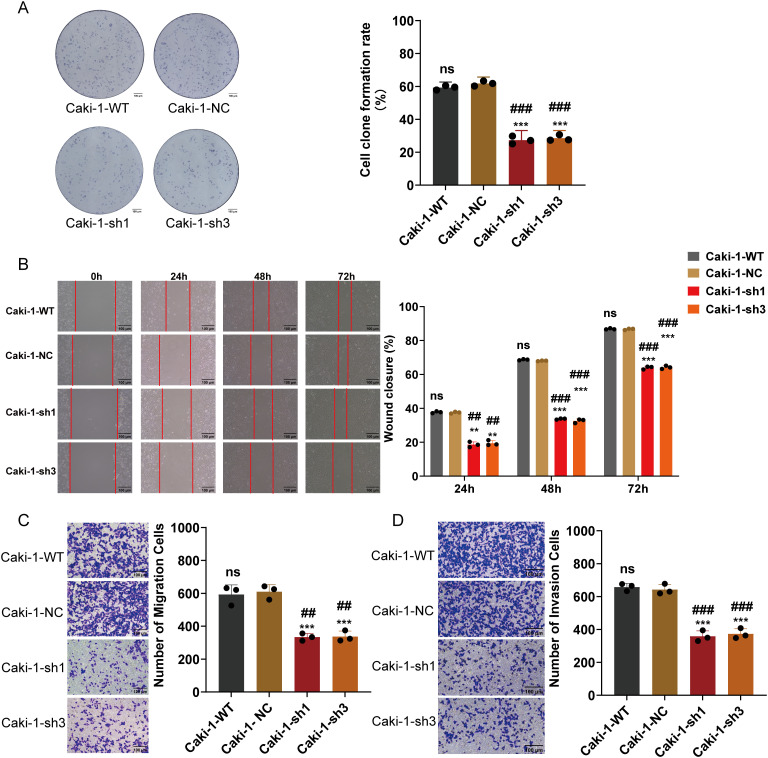
Functional effects of *ARPC1B* silencing on Caki-1 cells. (**A**) Reduced clonogenic potential of Caki-1 cells after ARPC1B knockdown. (**B**) Inhibition of migration observed in Caki-1 cells upon ARPC1B silencing (WHA). (**C,D**) Reduced migration and invasion capabilities demonstrated by Transwell assays following ARPC1B knockdown. Compared to the sh-NC group, **p < 0.01, ***p < 0.001, ns, *p* **> **0.05. Compared to the WT group, ^##^*p* < 0.01, ^###^*p* < 0.001

To elucidate the biological impact of reduced ARPC1B expression, various cellular functional assays were conducted, focusing on ccRCC cellular behaviors. Cell proliferation assays using the CCK-8 method revealed a significant suppression of growth rate in ARPC1B-silenced 786-O and Caki-1 cells (*p* < 0.001; Fig. 3A,B), confirming ARPC1B’s supportive role in cellular proliferation. Colony formation assays further illustrated significantly impaired clonogenic capacity in cells lacking ARPC1B expression relative to controls (*p* < 0.001; Figs. 3C, A4A), underscoring ARPC1B’s relevance to cellular survival and colony-forming abilities. Additionally, wound-healing assays demonstrated significantly reduced migratory abilities in ARPC1B-depleted cells over 72 h in comparison with WT and NC groups (*p* < 0.05; Figs. 3D, A4B). Correspondingly, Transwell-based migration and invasion assays showed notably diminished invasive and migratory capacities in ARPC1B-knockdown groups compared to controls (*p* < 0.001; Figs. 3E,F, A4C,D). Collectively, these results substantiate that ARPC1B depletion prominently diminishes proliferation, clonogenicity, migratory potential, and invasiveness in ccRCC cell lines.

### 3.4 ARPC1B Overexpression Promotes Proliferation, Colony Formation, Migration, and Invasion of ccRCC Cells In Vitro

**Figure 4:**
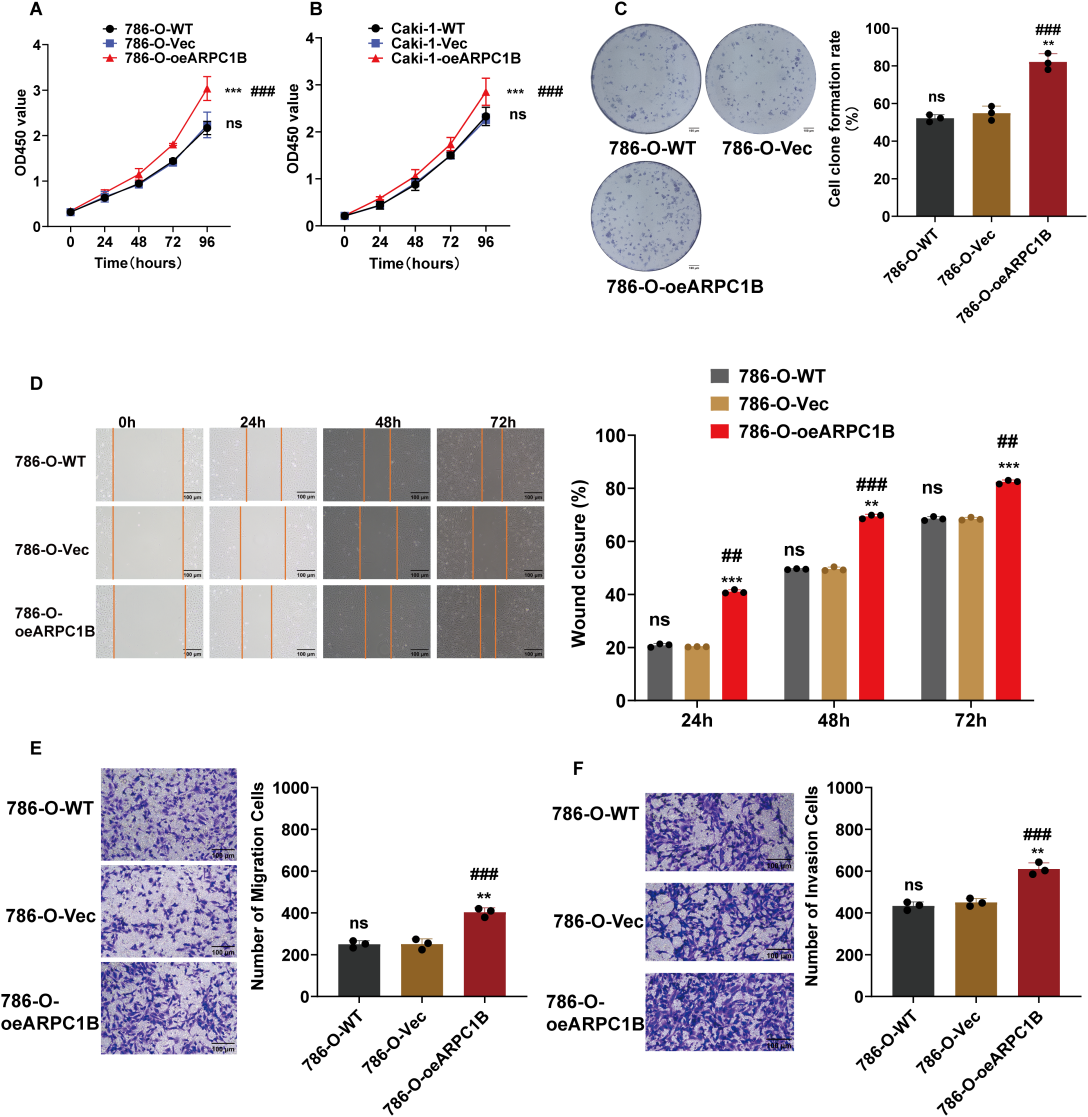
Effect of ARPC1B overexpression on ccRCC cell behavior. (**A,B**) Increased cell proliferation rates in ARPC1B-overexpressing 786-O and Caki-1 cells measured by CCK-8 assays. (**C**) Enhanced colony formation ability of 786-O cells following ARPC1B overexpression. (**D**) Accelerated migration observed in ARPC1B-overexpressing cells by WHA. (**E,F**) Transwell assays demonstrating increased migration and invasion capabilities in ARPC1B-overexpressing 786-O cells. Control group: Vec (GV492-empty). Compared to the Vec group; **p < 0.01, ***p < 0.001, ns, *p* **> **0.05. Compared to the WT group; ^##^*p* < 0.01, ^###^*p* < 0.001

**Figure A5:**
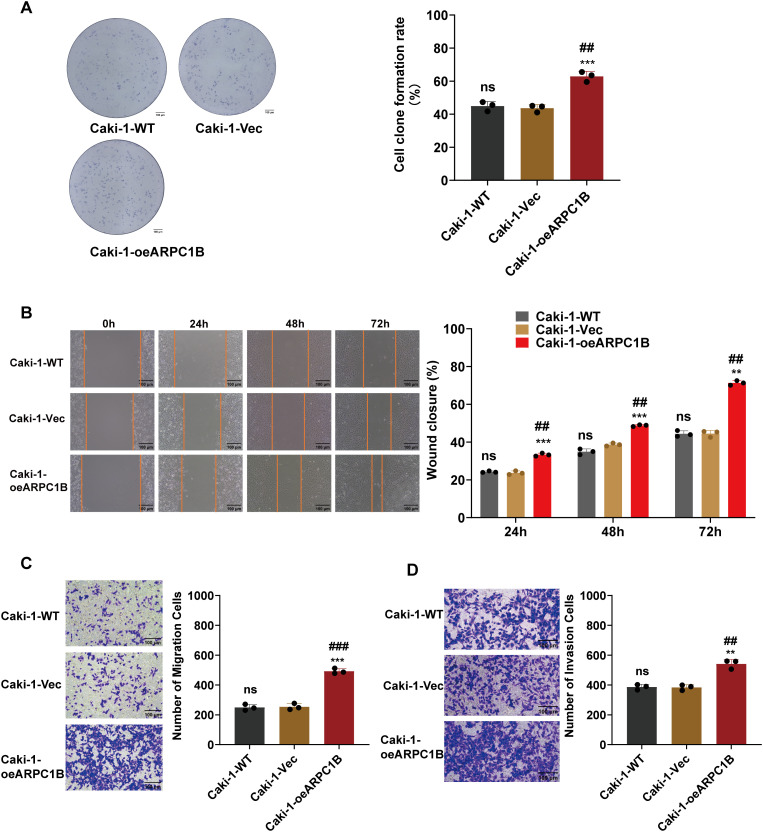
Effect of *ARPC1B* overexpression on Caki-1 cell behavior. (**A**) Enhanced colony formation ability of Caki-1 cells following ARPC1B overexpression. (**B**) Accelerated migration observed in ARPC1B-overexpressing cells by WHA. (**C,D**) Transwell assays demonstrating increased migration and invasion capabilities in ARPC1B-overexpressing Caki-1 cells. Control group: Vec (GV492-empty). Compared to the Vec group, **p < 0.01, ***p < 0.001, ns, *p* **> **0.05. Compared to the WT group; ^##^*p* < 0.01, ^###^*p* < 0.001

To further confirm ARPC1B’s oncogenic activity, functional assays were conducted on 786-O and Caki-1 cells stably overexpressing ARPC1B. CCK-8 proliferation assays showed significantly increased proliferation rates in ARPC1B-overexpressing cells compared to WT and empty vector control groups (*p* < 0.001; Fig. 4A,B). Colony formation assays similarly revealed an elevated number and size of colonies in ARPC1B-overexpressing cells vs. control cells (*p* < 0.01; Figs. 4C, A5A), reflecting the enhanced long-term growth potential conferred by ARPC1B. WHAs indicated significantly accelerated wound closure rates in ARPC1B-overexpressing 786-O and Caki-1 cells relative to controls (*p* < 0.01; Figs. 4D, A5B). Moreover, Transwell assays demonstrated that ARPC1B-overexpressing cells displayed significantly enhanced migration and invasion activities compared with WT and vector controls (*p* < 0.001; Figs. 4E,F, A5C,D), further validating ARPC1B’s role in promoting ccRCC metastatic properties. Collectively, these results underscore that ARPC1B upregulation enhances proliferation, colony formation, migration, and invasion capacities of ccRCC cells.

### 3.5 ARPC1B Knockdown Inhibits Tumor Growth In Vivo

**Figure 5:**
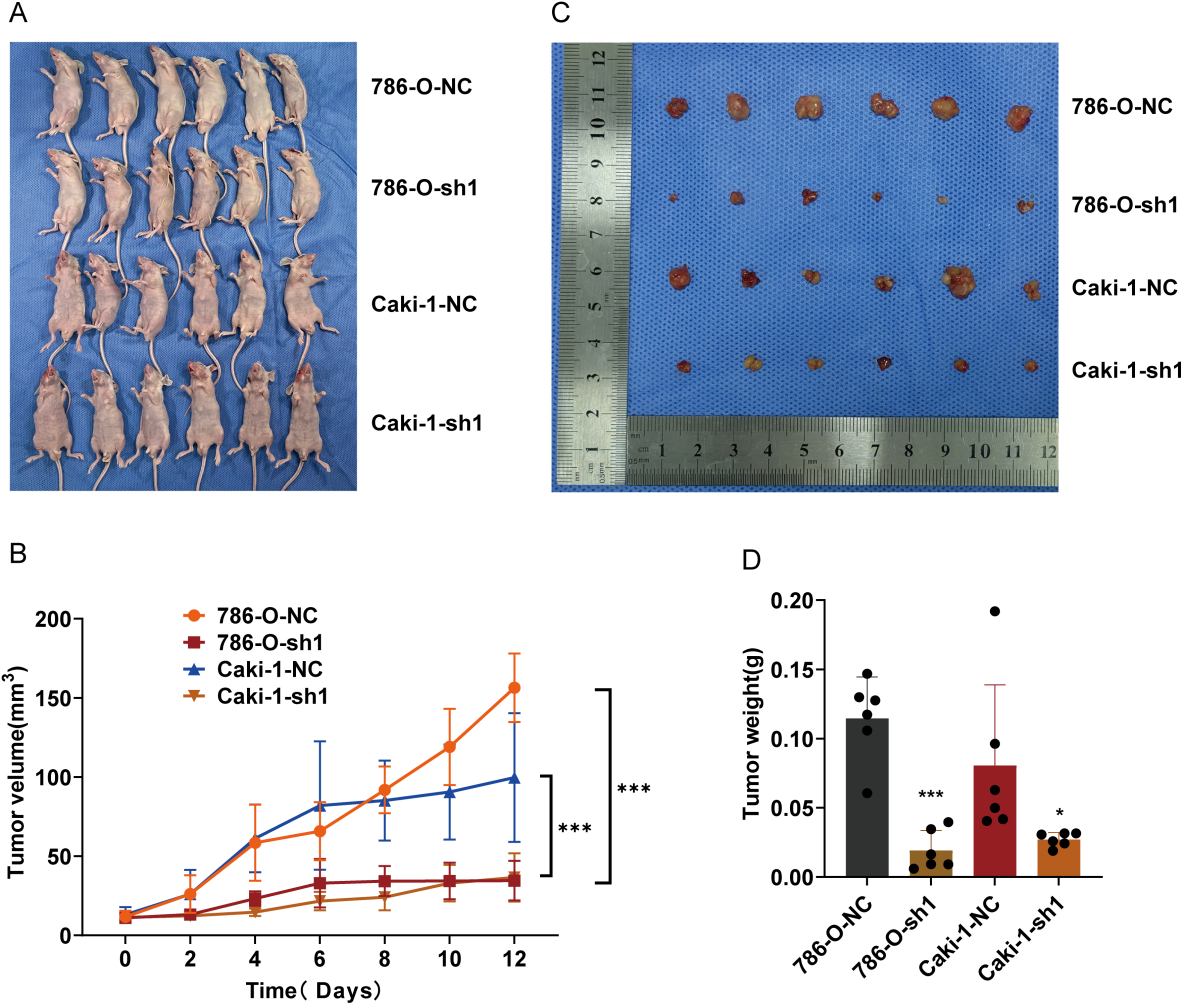
Influence of ARPC1B knockdown on ccRCC tumor growth *in vivo*. (**A**) Representative images of nude mice bearing xenografts. (**B**) Images of excised ccRCC xenografts. (**C**) Tumor volume curves over time. (**D**) Comparison of tumor weights. Compared to the sh-NC group; *p < 0.05, ***p < 0.001

**Figure A6:**
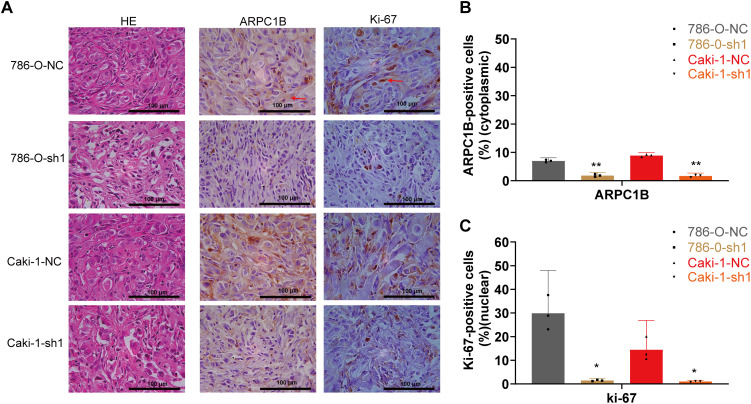
Effects of *ARPC1B* knockdown on tumor growth *in vivo*. (**A**) Representative images of HE stained and IHC stained xenografts. Red arrows were used to highlight the positive area. (**B**) Quantification of cytoplasmic ARPC1B-positive cells. (**C**) Quantification of nuclear Ki-67-positive cells. Compared to the sh-NC group; *p < 0.05, **p < 0.01

To assess ARPC1B’s functional role *in vivo*, tumor xenografts were established in nude mice via subcutaneous injection of ARPC1B-knockdown or NC cells (786-O and Caki-1). Tumor volume monitoring over 12 days revealed significantly suppressed tumor growth in ARPC1B-knockdown groups (*p* < 0.001, Fig. 5A,B). Representative images confirmed smaller tumor sizes upon ARPC1B silencing (Fig. 5C). At the conclusion of experiments, tumor weights were significantly lower in ARPC1B-knockdown groups relative to NC groups (*p* < 0.001 for 786-O; *p* < 0.05 for Caki-1; Fig. 5D). Histological evaluation using hematoxylin and eosin (H&E) staining indicated lower cellular density and less aggressive tumor morphology in ARPC1B-knockdown xenografts (Fig. A6). Immunohistochemistry (IHC) further demonstrated reduced ARPC1B expression and diminished Ki-67 staining in ARPC1B-silenced tumors, indicating decreased tumor cell proliferation (Fig. A6). Taken together, these findings strongly support that ARPC1B knockdown significantly reduces tumor growth, tumor mass, and proliferation *in vivo*, highlighting its critical contribution to ccRCC progression.

### 3.6 ARPC1B Promotes ccRCC Progression via EMT In Vitro and In Vivo

**Figure 6:**
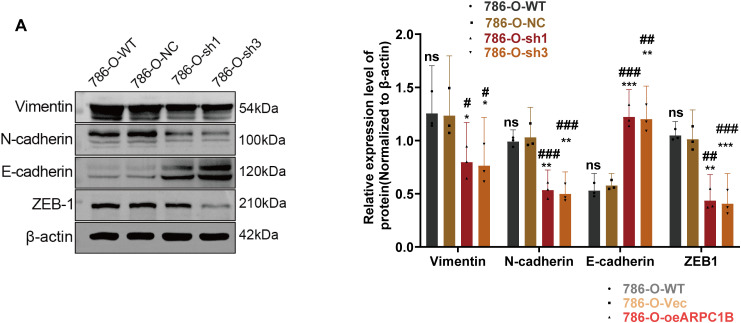

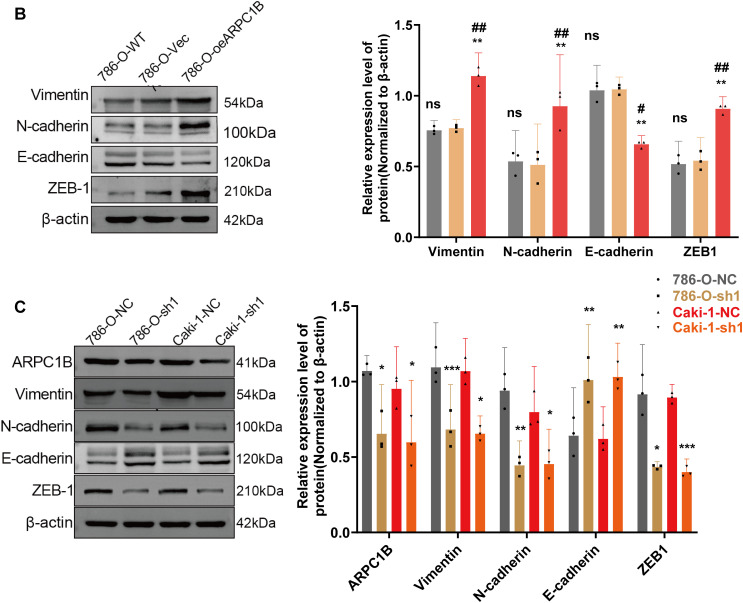
Relationship between ARPC1B expression and EMT markers *in vitro* and *in vivo*. (**A**) WB analysis of EMT markers (Vimentin, N-cadherin, ZEB-1, and E-cadherin) in ARPC1B-silenced 786-O cells. (**B**) Protein expression of EMT markers in 786-O cells with ARPC1B overexpression. (**C**) WB assessment of EMT marker expression in xenograft tumors from ARPC1B-knockdown groups. All samples were analyzed simultaneously under consistent conditions, and were normalized to β-actin. Compared to the sh-NC group, Vec group; *p < 0.05, **p < 0.01, ***p < 0.001, ns, *p* **>** 0.05. Compared to the WT group; ^#^*p* < 0.05, ^##^*p* < 0.01, ^###^*p* < 0.001

**Figure A7:**
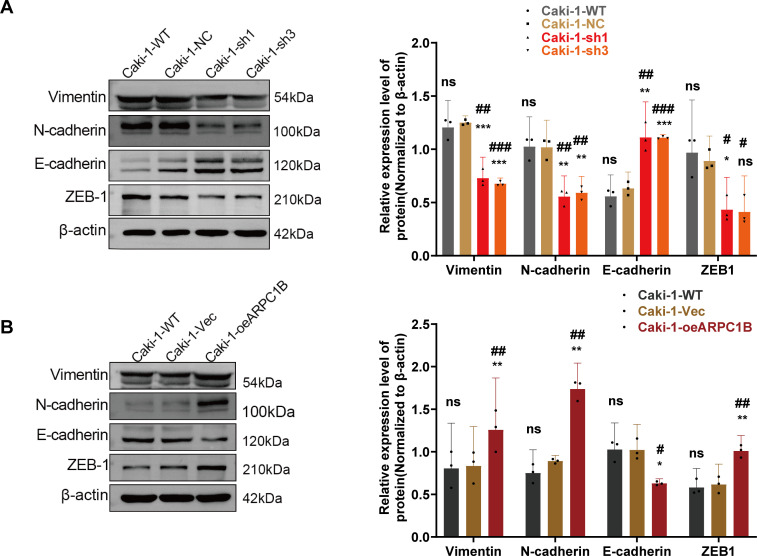

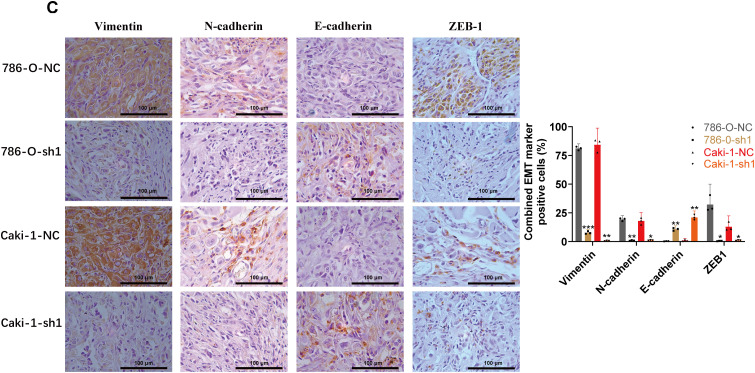
Relationship between *ARPC1B* expression and EMT markers *in vitro* and *in vivo*. (**A**) WB analysis of EMT markers (Vimentin, N-cadherin, ZEB-1, and E-cadherin) in ARPC1B-silenced Caki-1 cells. (**B**) Protein expression of EMT markers in Caki-1 cells with *ARPC1B* overexpression. (**C**) IHC examination of EMT marker expression in xenograft tumors from ARPC1B-knockdown groups. All samples were analyzed simultaneously under consistent conditions, and were normalized to β-actin. Compared to the sh-NC group; *p < 0.05, **p < 0.01, ***p < 0.001, ns, *p* **> **0.05. Compared to the WT group, ^#^*p* < 0.05, ^##^*p* < 0.01, ^###^*p* < 0.001

EMT is essential for facilitating tumor cell invasion and metastasis [35–37]. To determine whether ARPC1B affects ccRCC invasiveness by modulating EMT processes, EMT-related markers were analyzed via WB. In 786-O and Caki-1 cells, suppression of ARPC1B significantly diminished mesenchymal marker proteins, including Vimentin, N-cadherin, and ZEB-1, while concurrently increasing the epithelial marker E-cadherin (*p* < 0.05; Figs. 6A, A7A). These data suggest that loss of ARPC1B expression inhibits EMT. Conversely, cells with enhanced ARPC1B expression showed marked increases in the levels of Vimentin, N-cadherin, and ZEB-1 proteins, alongside a decline in E-cadherin expression (*p* < 0.05; Figs. 6B, A7B), indicating that ARPC1B overexpression promotes EMT, thereby facilitating cell invasion. To substantiate these findings *in vivo*, xenograft tumors established from 786-O and Caki-1 cells with ARPC1B knockdown were examined. WB assays demonstrated decreased expression of mesenchymal proteins coupled with elevated E-cadherin levels in ARPC1B-deficient tumors compared with negative control (NC) tumors (Fig. 6C). IHC analysis corroborated these findings, with reduced staining intensity for mesenchymal markers (Vimentin, N-cadherin, ZEB-1) and increased intensity for E-cadherin observed in ARPC1B-knockdown tumor tissues (Fig. A7C). Collectively, these observations imply that ARPC1B enhances ccRCC migration and invasion through EMT induction.

### 3.7 ARPC1B Promotes ccRCC Progression via Wnt/β*-Catenin* Signaling In Vitro and In Vivo

**Figure 7:**
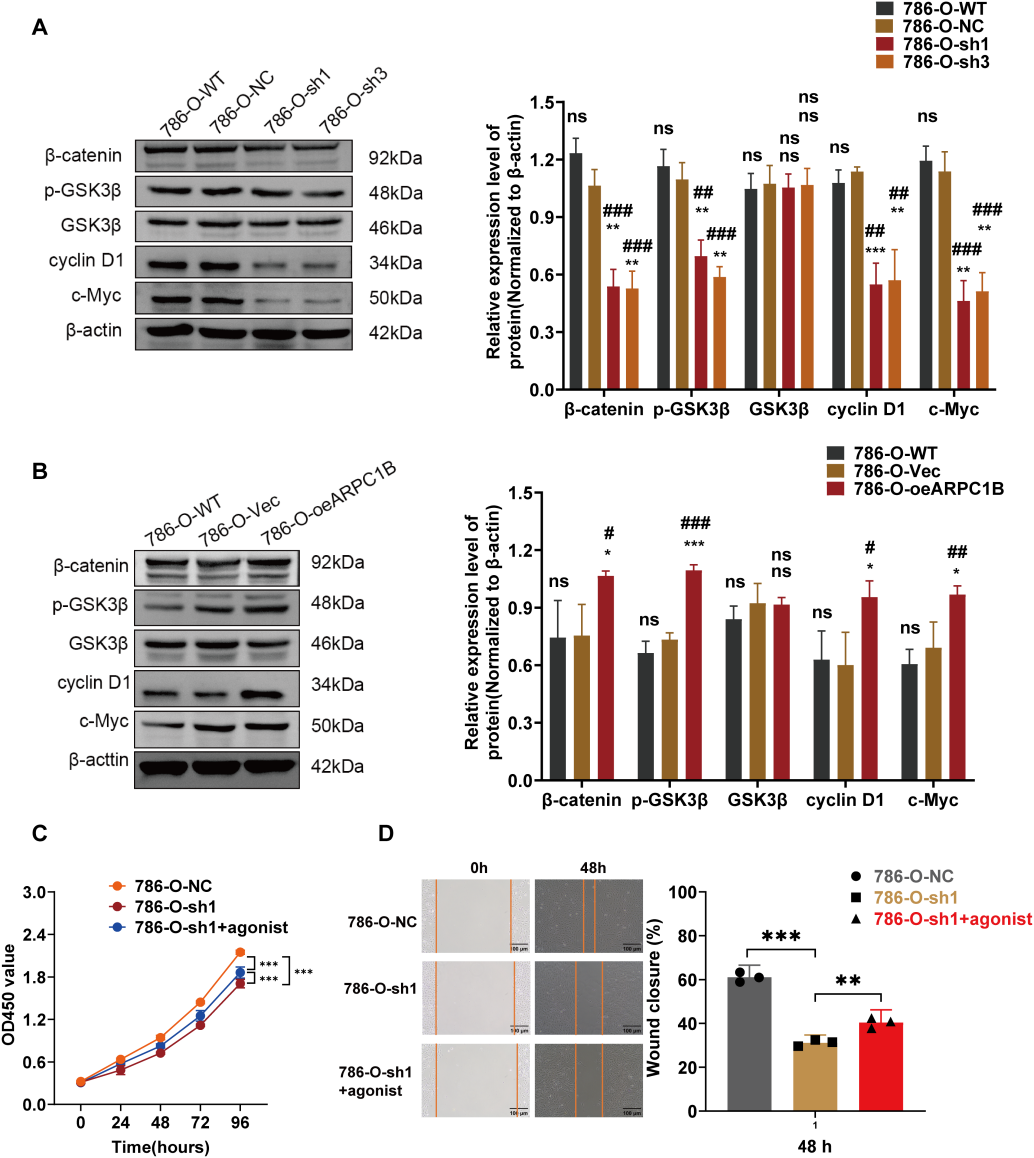
Correlation between ARPC1B expression and Wnt/β-catenin signaling. (**A**) WB analysis of β-catenin, p-GSK3β, GSK3β, c-Myc, and cyclin D1 expression in 786-O cells following ARPC1B knockdown. (**B**) WB assessment of β-catenin, p-GSK3β, GSK3β, c-Myc, and cyclin D1 protein levels in ARPC1B-overexpressing 786-O cells. All samples were analyzed simultaneously under consistent conditions, and were normalized to β-actin. (**C, D**) Rescue experiments examining proliferation (CCK-8 assay) and migration (WHA) in ARPC1B-silenced cells treated with Wnt/β-catenin agonist 3. Compared to the sh-NC group, Vec group, sh1 group; *p < 0.05, **p < 0.01, ***p < 0.001, ns, *p* **> **0.05. Compared to the WT group; ^#^*p* < 0.05, ^##^*p* < 0.01, ^###^*p* < 0.001, ns, *p* **> **0.05

**Figure 8:**
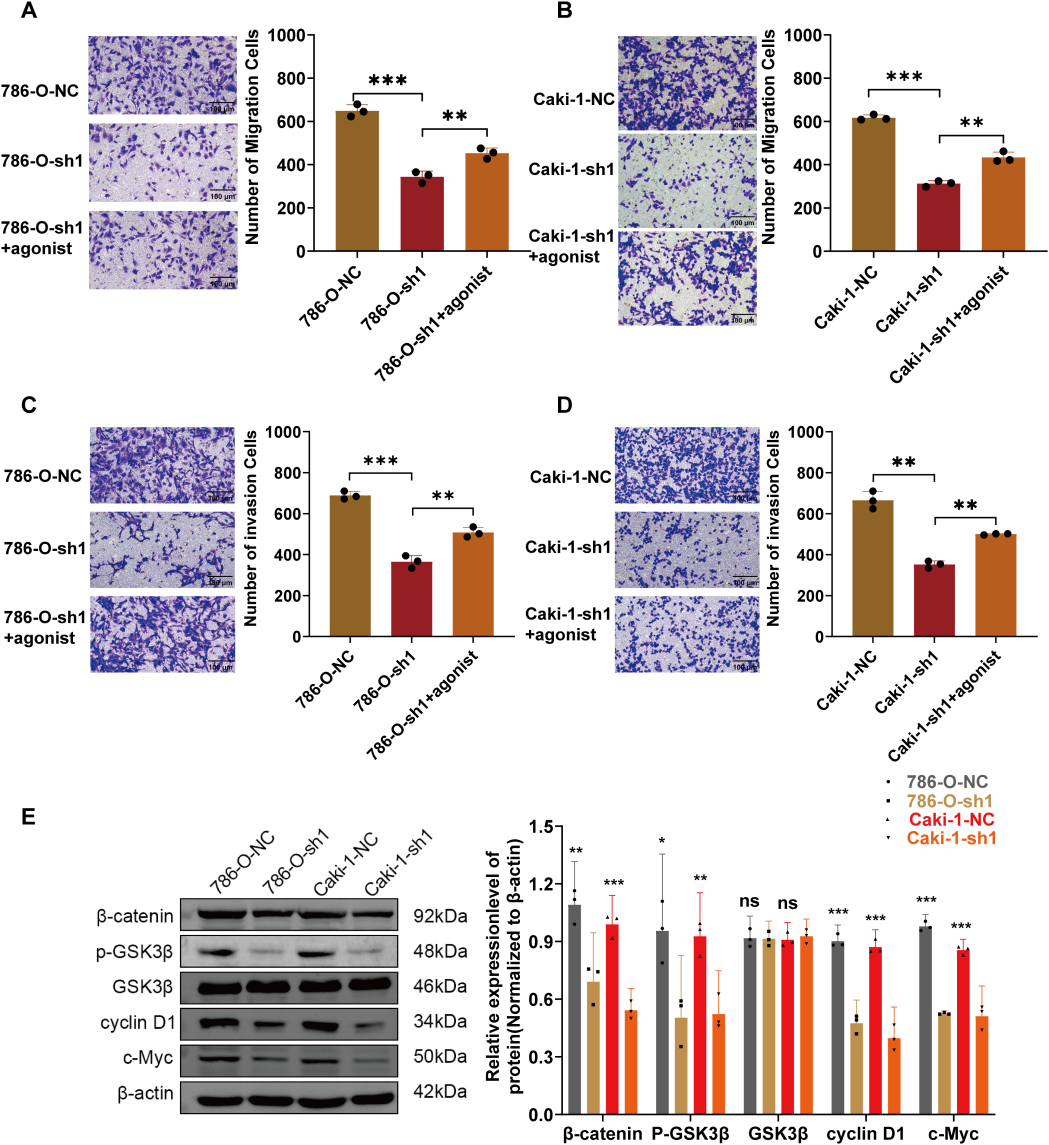
Validation of ARPC1B involvement in Wnt/β-catenin pathway activation. (**A–D**) Rescue experiments evaluating migration and invasion capabilities in ARPC1B-knockdown cells treated with Wnt/β-catenin agonist 3. (**E**) WB analysis of β-catenin, p-GSK3β, GSK3β, c-Myc, and cyclin D1 protein expression in xenograft tumor tissues derived from ARPC1B-silenced cells. All samples were analyzed simultaneously under consistent conditions, and were normalized to β-actin. Compared to the sh1 group; *p < 0.05, **p < 0.01, ***p < 0.001, ns, *p* **> **0.05

**Figure A8:**
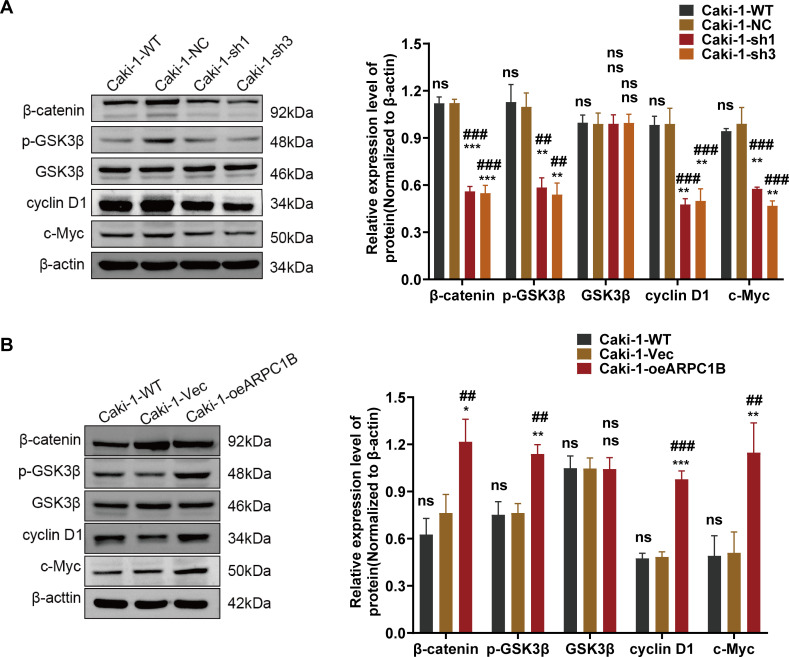

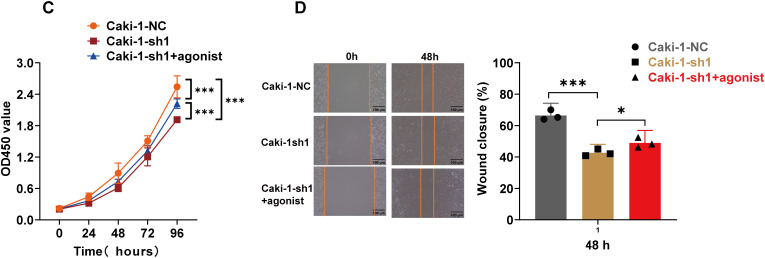
Correlation between *ARPC1B* expression and Wnt/β-catenin signaling. (**A**) WB analysis of β-catenin, p-GSK3β, GSK3β, c-Myc, and cyclin D1 expression in Caki-1 cells following ARPC1B knockdown. (**B**) WB assessment of β-catenin, p-GSK3β, GSK3β, c-Myc, and cyclin D1 protein levels in ARPC1B-overexpressing Caki-1 cells. All samples were analyzed simultaneously under consistent conditions, and were normalized to β-actin. (**C,D**) Rescue experiments examining proliferation (CCK-8 assay) and migration (WHA) in ARPC1B-silenced cells treated with Wnt/β-catenin agonist 3. Compared to the sh-NC group, Vec group; *p < 0.05, **p < 0.01, ***p < 0.001, ns, *p* **> **0.05. Compared to the WT group, ^##^*p* < 0.01, ^###^*p* < 0.001, ns, *p* **> **0.05

**Figure A9:**
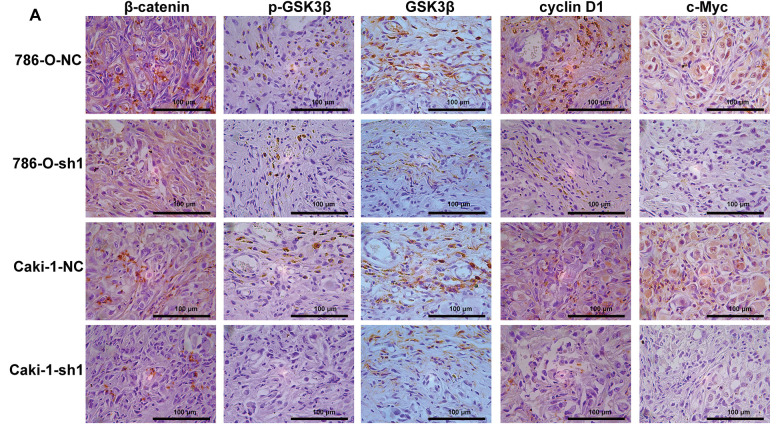

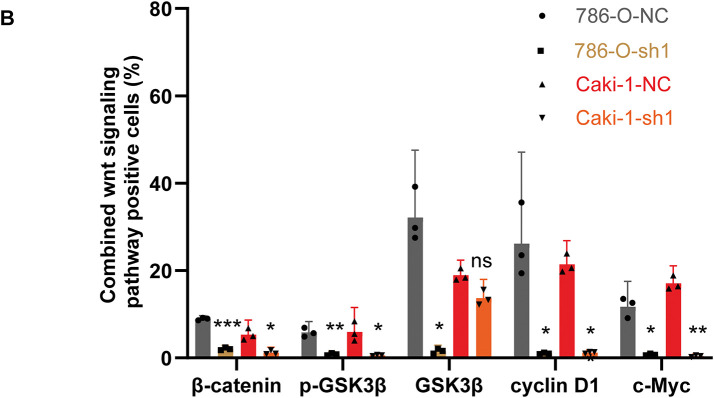
IHC examination of Wnt/β-catenin signaling marker expression in xenograft tumors from ARPC1B-knockdown groups. (**A**) Representative images of IHC stained xenografts. (**B**) Quantification of Wnt/β-catenin signaling marker positive cells (%). Compared to the sh-NC group; *p < 0.05, **p < 0.01, ***p < 0.001, ns, *p* **> **0.05

Prior research has demonstrated that ARPC1B facilitates ovarian cancer progression via activation of the Wnt/β-catenin cascade [30]. Consequently, this study hypothesized a parallel regulatory role of ARPC1B in ccRCC progression. WB analyses revealed significant decreases in protein levels of β-catenin, phosphorylated GSK3β (p-GSK3β), c-Myc, and cyclin D1 in 786-O and Caki-1 cells following ARPC1B silencing (*p* < 0.01, Figs. 7A, A8A). Conversely, ARPC1B overexpression notably increased these signaling molecules (*p* < 0.05, Figs. 7B, A8B), confirming ARPC1B’s regulatory role in Wnt/β-catenin pathway activation. Rescue experiments using Wnt/β-catenin agonist 3 further validated this mechanistic link. Treatment with agonist 3 restored β-catenin, p-GSK3β, c-Myc, and cyclin D1 expression levels and reversed the inhibitory effects of ARPC1B knockdown on cell proliferation (*p* < 0.001, Figs. 7C, A8C), wound healing (*p* < 0.05, Figs. 7D, A8D), Transwell migration (Fig. 8A,B), and invasion (Fig. 8C,D) assays in both cell lines. These findings demonstrate ARPC1B-mediated tumor aggressiveness via Wnt/β-catenin signaling. Consistent with *in vitro* findings, xenograft tumor tissues from ARPC1B-knockdown groups exhibited significantly reduced protein levels of β-catenin, p-GSK3β, c-Myc, and cyclin D1 (*p* < 0.05, Fig. 8E). IHC analysis confirmed reduced staining of these proteins in ARPC1B-knockdown tumor tissues relative to NC controls (Fig. A9). Altogether, these results highlight ARPC1B as a critical regulator of ccRCC progression, exerting oncogenic effects through activation of the Wnt/*β*-catenin pathway.

## 4 Discussion

ccRCC ranks among the most frequent malignancies of the urinary system. Despite recent progress in targeted treatment strategies, patients with advanced or metastatic ccRCC continue to exhibit poor clinical outcomes [38,39]. Thus, clarifying the molecular mechanisms driving ccRCC progression remains crucial for identifying novel therapeutic targets. Recent investigations have underscored ARPC1B as a significant contributor to tumor progression, particularly in promoting invasion and metastasis, and associated its elevated expression with unfavorable prognoses in multiple cancer types [29,30,40]. Nevertheless, the precise functional significance of ARPC1B in ccRCC progression remains inadequately characterized.

*ARPC1B* emerges as a multi-dimensional regulator orchestrating ccRCC progression through synergistic control of metastatic dissemination and pathway activation. IHC profiling validated its clinical relevance, demonstrating strong correlations with advanced TNM stages (*p* = 0.003) and high Fuhrman grades (*p* < 0.001), alongside 2.9-fold tumor-specific overexpression (38% vs. 13% in paracancerous tissues, *p* = 0.009). Critically, multivariate analysis revealed ARPC1B’s independent prognostic value (Cox *p* = 0.009), surpassing conventional staging systems, establishing its utility as a stratification biomarker.

Clinically, the association between *ARPC1B* overexpression and lymph node metastasis (*p* = 0.029) underscores its relevance to advanced disease management. However, limited statistical power due to sample imbalance in lymph node-positive subgroups (3 vs. 147 cases) necessitates cautious interpretation. Future multicenter studies with enriched metastatic cohorts are required to validate *ARPC1B*’s role. Nevertheless, concordance between IHC profiles and the GEPIA database validation strengthens clinical data reliability. Independent functional coherence across 786-O and Caki-1 models further supports biological plausibility despite cohort constraints.

Through comprehensive functional analyses, this study established ARPC1B’s critical role in ccRCC progression. *In vitro* experiments indicated that ARPC1B significantly facilitates proliferation, migration, and invasion, while knockdown effectively suppresses these oncogenic processes. Corroborating these *in vitro* results, xenograft models demonstrated that ARPC1B depletion markedly decreased tumor growth, reinforcing its importance in tumor development. These findings align with prior research in ovarian cancer and glioblastoma, which similarly reported ARPC1B’s capacity to enhance tumor cell proliferation and invasive behavior [30,40].

At a mechanistic level, our data underscore that ARPC1B promotes ccRCC advancement through stimulation of EMT, an essential mechanism underlying tumor invasion and metastatic capability [35–37]. Specifically, depletion of ARPC1B led to decreased mesenchymal markers (N-cadherin, Vimentin, and ZEB-1) alongside elevated epithelial marker E-cadherin, indicative of EMT inhibition. Conversely, elevated ARPC1B expression strongly upregulated EMT markers, affirming ARPC1B’s crucial role in facilitating tumor cell invasiveness. Additionally, our investigation identified the Wnt/β-catenin pathway as a key downstream effector regulated by ARPC1B in ccRCC. Suppression of ARPC1B resulted in reduced expression of pivotal signaling proteins, including β-catenin, p-GSK3β, c-Myc, and cyclin D1, whereas ARPC1B overexpression enhanced their levels. Rescue experiments with Wnt/β-catenin agonist 3 reversed the inhibitory effects caused by ARPC1B knockdown, thus establishing a clear functional linkage between ARPC1B and Wnt/β-catenin signaling. These observations align with earlier reports suggesting that ARPC1B stabilizes β-catenin, enhancing its oncogenic signaling activities [41–43].

Nevertheless, the present study has several limitations. In particular, the relatively small clinical cohort and lack of genetically engineered animal models may restrict the generalizability of these conclusions. Furthermore, although we have demonstrated the activation of EMT and the Wnt/β-catenin pathway by ARPC1B, the precise molecular interactions through which ARPC1B modulates key proteins such as β-catenin and N-cadherin still require further detailed investigation. Subsequent research will employ techniques such as proximity-dependent biotin identification (BioID) and co-immunoprecipitation (co-IP) to systematically identify ARPC1B-binding proteins and further elucidate their precise functional roles in these signaling networks.

Collectively, our findings provide compelling evidence highlighting ARPC1B as a vital regulator of ccRCC tumor progression. Elevated ARPC1B expression correlates significantly with aggressive tumor phenotypes, poor clinical outcomes, and activation of key oncogenic signaling pathways, specifically EMT and Wnt/β-catenin. Therapeutic targeting of *ARPC1B* in ccRCC is mechanistically supported by preclinical evidence from glioblastoma, where its modulation enhances radiotherapy sensitivity and synergizes with immune checkpoint inhibitors to augment antitumor immunity [40,44]. Given ARPC1B’s role in cytoskeletal dynamics and metastasis suppression, combining its inhibition with immune checkpoint blockade represents a promising strategy for metastatic ccRCC that may improve survival outcomes. This approach, however, presents dual therapeutic challenges: ARPC1B deficiency compromises cytotoxic T lymphocyte effector functions as demonstrated by impaired tumor cell killing and attenuated IFN-γ secretion in standardized systems [25], while close structural homology with *ARPC1A* raises toxicity and specificity concerns [45]. Further research remains essential to delineate *ARPC1B’*s role in ccRCC and advance its application in precision oncology.

## Data Availability

The data that support the findings of this study are available from the Corresponding Authors, upon reasonable request.

## Abbreviations

ARPC1B: Actin-Related Protein 2/3 Complex Subunit 1B
ANOVA: Analysis of Variance
ccRCC: Clear Cell Renal Cell Carcinoma
cDNA: Complementary DNA
CT: Threshold Cycle
CI: Confidence Interval
EMT: Epithelial-Mesenchymal Transition
ECL: Enhanced Chemiluminescence
GEPIA: Gene Expression Profiling Interactive Analysis
HRP: Horseradish Peroxidase
IHC: Immunohistochemistry
lncRNAs: Long non-coding RNAs
NC: Negative Control
OS: Overall Survival
ORF: Open Reading Frame
OD: Optical Density
PBS: Phosphate-buffered Saline
*p-GSK3β*: *Phosphorylated GSK3β*
RIPA: Radioimmunoprecipitation Assay
shRNA: Short Hairpin RNA
TMA: Tissue Microarray
TBST: Tris-buffered Saline with Tween^®^-20
RT-qPCR: Tissue Microarray Reverse Transcription Quantitative Polymerase Chain Reaction
WB: Western blotting
WT: Wild-Type
